# Supplementary material for: The WOPR family protein Ryp1 is a key regulator of gene expression, development, and virulence in the thermally dimorphic fungal pathogen Coccidioides posadasii
Source: PLoS Pathog. 2022 Apr 6;18(4):e1009832. doi: 10.1371/journal.ppat.1009832 (PMC9015156; doi:10.1371/journal.ppat.1009832)
Supplement: S3 Table — (DOCX) [file ppat.1009832.s003.docx]

**S3 Table.** Oligonucleotides used for ∆*ryp1* mutant creation and analysis.

| **Primer** | **Purpose** | **Sequence** |
| --- | --- | --- |
| OAM431 | *hphB* oligo for transformant analysis | GACCTCCACTAGCTCCAGCCAAGCC |
| OAM432 | *hphB* oligo for transformant analysis | GATAGTGGAAACCGACGCCCCAGCAC |
| OAM597 | *hphB* gene forward | CGACGTTAACTGATATTGAAGGAGC |
| OAM598 | *hphB* gene reverse | GTTAACTGGTTCCCGGTCGGCATCT |
| OAM1153 | *RYP1* 5’ flank | GCTGGTCTCGAACGGAGCGCTTAGC |
| OAM1154 | *RYP1* 5’ flank and Hph 5’ | GCTCCTTCAATATCAGTTAACGTCGAAAAGTAGCAGATAAAGACCGCAGCG |
| OAM1155 | *RYP1* 3’ flank 3’ end | TAAACAGATTCGAGGAAAACGGCAC |
| OAM1156 | *RYP1* 3’Hph and 3’ flank, 5’ end | AGATGCCGACCGGGAACCAGTTAACACAACCACCTATGCAGCACACATCCC |
| OAM1159 | Nested primer 5’ end | GGGGGATCCAGAGGCCGGGTTTCTCTTCGTGTGA |
| OAM1160 | Nested primer 3’ end | CCCGGATCCATCATGCGTATTACCATCAGAGAAA |
| OAM1611 | 5’ *RYP1* primer for transformant analysis | GCTGGTCTCGAACGGAGCGC |
| OAM1612 | 3’ *RYP1* primer for transformant analysis | GACACACTTAAACAGATTCGAGG |
